# A systematic review and meta-analysis comparing the diagnostic capability of automated breast ultrasound and contrast-enhanced ultrasound in breast cancer

**DOI:** 10.3389/fonc.2023.1305545

**Published:** 2024-01-09

**Authors:** Haoyu Zhang, Jingyi Hu, Rong Meng, Fangfang Liu, Fan Xu, Min Huang

**Affiliations:** ^1^ Department of Clinic Medicine, Chengdu Medical College, Sichuan, China; ^2^ Department of Public Health, Chengdu Medical College, Sichuan, China; ^3^ Art College, Southwest Minzu University, Sichuan, China; ^4^ Department of Physiology, School of Basic Medicine, Chengdu Medical College, Sichuan, China

**Keywords:** breast cancer, automated breast ultrasound (ABUS), contrast-enhanced ultrasound (CEUS), diagnosis, meta-analysis

## Abstract

**Objective:**

To compare the diagnostic performance of automated breast ultrasound (ABUS) and contrast-enhanced ultrasound (CEUS) in breast cancer.

**Methods:**

Published studies were collected by systematically searching the databases PubMed, Embase, Cochrane Library and Web of Science. The sensitivities, specificities, likelihood ratios and diagnostic odds ratio (DOR) were confirmed. The symmetric receiver operator characteristic curve (SROC) was used to assess the threshold of ABUS and CEUS. Fagan’s nomogram was drawn. Meta-regression and subgroup analyses were applied to search for sources of heterogeneity among the included studies.

**Results:**

A total of 16 studies were included, comprising 4115 participants. The combined sensitivity of ABUS was 0.88 [95% CI (0.73–0.95)], specificity was 0.93 [95% CI (0.82–0.97)], area under the SROC curve (AUC) was 0.96 [95% CI (0.94–0.96)] and DOR was 89. The combined sensitivity of CEUS was 0.88 [95% CI (0.84–0.91)], specificity was 0.76 [95% CI (0.66–0.84)], AUC was 0.89 [95% CI (0.86–0.92)] and DOR was 24. The Deeks’ funnel plot showed no existing publication bias. The prospective design, partial verification bias and blinding contributed to the heterogeneity in specificity, while no sources contributed to the heterogeneity in sensitivity. The post-test probability of ABUS in BC was 75%, and the post-test probability of CEUS in breast cancer was 48%.

**Conclusion:**

Compared with CEUS, ABUS showed higher specificity and DOR for detecting breast cancer. ABUS is expected to further improve the accuracy of BC diagnosis.

## Introduction

Having displaced lung cancer, breast cancer (BC) has become the most frequently diagnosed cancer across the globe and accounts for 1 in 8 of all cancer diagnoses (1). Until 2020, there had been over 2.3 million new cases and 685,000 deaths in BC patients globally (1). Furthermore, the treatment of patients with advanced BC is difficult, and the cure rate is low (2, 3). The relative survival of patients diagnosed with early-stage BC is much higher than that of patients diagnosed with late-stage disease. The 5-year relative survival for BC patients is >99% for stage I disease, 93% for stage II, 75% for stage III, and 29% for stage IV (4). Therefore, early detection, early diagnosis and early treatment are the keys to reducing the mortality rate and improving the prognosis of breast cancer.

Mammography (MG), as the main method of BC screening and diagnosis, has been recognized by most clinicians and radiologists. However, for dense breasts, MG has low sensitivity and specificity, a high probability of false-negative results, uses ionizing radiation and has other shortcomings (5). Ultrasonography has become an important auxiliary imaging method for the diagnosis of breast diseases MG (6). HHUS (handheld ultrasound) has become the most used ultrasound method for the evaluation of breast diseases due to its convenience, high resolution and absence of ionizing radiation (7). However, HHUS has disadvantages, such as a high operator dependence and real-time diagnosis.

To reduce operator dependence, automated breast ultrasound has been developed. Automated breast ultrasound (ABUS) has many advantages over conventional ultrasound. ABUS enables visualization from the skin surface on the breast to the thoracic wall and reserves all the breast volume information on a picture archiving and communication system (8). ABUS has similar diagnostic quality to hand-held ultrasonography in screening. Nevertheless, it can assess the location and size of masses more accurately than HHUS (8).

Contrast-enhanced ultrasound (CEUS) is a pure blood pool imaging technology, which can not only display the morphology of breast lesions but also evaluate the morphology and dynamics of the blood supply to the lesions. Compared with that of US, it has been confirmed that the diagnostic efficiency of CEUS is higher (9). CEUS improves backscattering in the vascular system by injecting contrast agents (gas-filled microbubbles). Therefore, sonographers can make out certain vascular structures and tissues that differ in vascularity in the masses, whereupon one can analyse breast lesions features quantitatively and qualitatively (8).

However, researchers differ in their understanding of the value of ABUS and CEUS in the diagnosis of breast cancer. The diagnostic capability of ABUS and CEUS in BC remains unclear. Therefore, this study evaluated and compared the diagnostic capability of ABUS and CEUS.

## Methods

### Search strategy

Two reviewers (ZHY and HJY) independently searched the PubMed, Embase, Cochrane Library and Web of Science databases up to April 2023. The search terms are shown below (Breast Neoplasm OR Breast Tumors OR Breast Cancer OR Malignant Neoplasm of Breast) AND (automated breast volume scan OR automatically generated breast volume scan OR ABVS OR contrast-enhanced ultrasound OR CEUS).

### Inclusion and exclusion criteria

The inclusion criteria included the following items: (1) well-defined BC patients included as study subjects; (2) randomized controlled trials divided into two groups, the experimental group with BC patients and the control group using patients with benign lesions; (3) clinical trials involving ABVS or/and CEUS for BC detection; (4) true-positive (TP), false-negative (FN), false-positive (FP), true-negative (TN), sensitivity (Se) and specificity (Sp) shown or figured out according to the literature; and (5) histological examination applied as the gold standard method of diagnosis. The exclusion criteria included following items: (1) animal studies; (2) non-case–control trials; (3) studies without sufficient or experimental data; (4) letters, case reports, guidelines, reviews, and conference abstracts; (5) literature published repeatedly; and (6) studies unrelated to diagnostic means in BC patients.

### Data extraction

Two investigators (ZHY and HJY) independently screened the demographic and intervention information from original studies. The extracted information and data were as follows: (1) name of the first author; (2) type of study; (3) region of the author; (4) sample size or number of lesions; (5) age and female/male ratio of experimental participants; (6) year the study was released; (7) gold standard used and (8) the outcome indicators of ABVS and CEUS, including TP, FP, NP, TN, Sp, Se etc.

### Statistical analysis

Stata 15.0 software (Stata Corp 4905 Lakeway Drive, TX, USA) was used to compare the diagnostic modalities in studies included in the meta-analysis. The bivariate model was applied to calculate combined sensitivity, specificity, the positive/negative likelihood ratio (PLR/NLR) and the diagnostic odds ratio (DOR). The area under the receiver operator characteristic (ROC) curve estimated the total diagnostic efficacy of ABVS or CEUS in BC patients. Post-test probability could determine whether the diagnostic probability was increased or decreased in comparison with the pre-test probability, which was assessed from conventional data, trial data or clinical decisions. The statistical heterogeneity based on the included studies was evaluated using the I^2^ statistics and Q test. Values of *I* ^2^ < 50% and *P* > 0.1 indicated what could be regarded as inhomogeneity, so a random-effects model was applied for further analysis. Otherwise, a fixed-effect model should be performed. A *P* value <0.05 indicated a significant difference between samples.

## Results

### Flow chart and study quality

**Figure 1:**
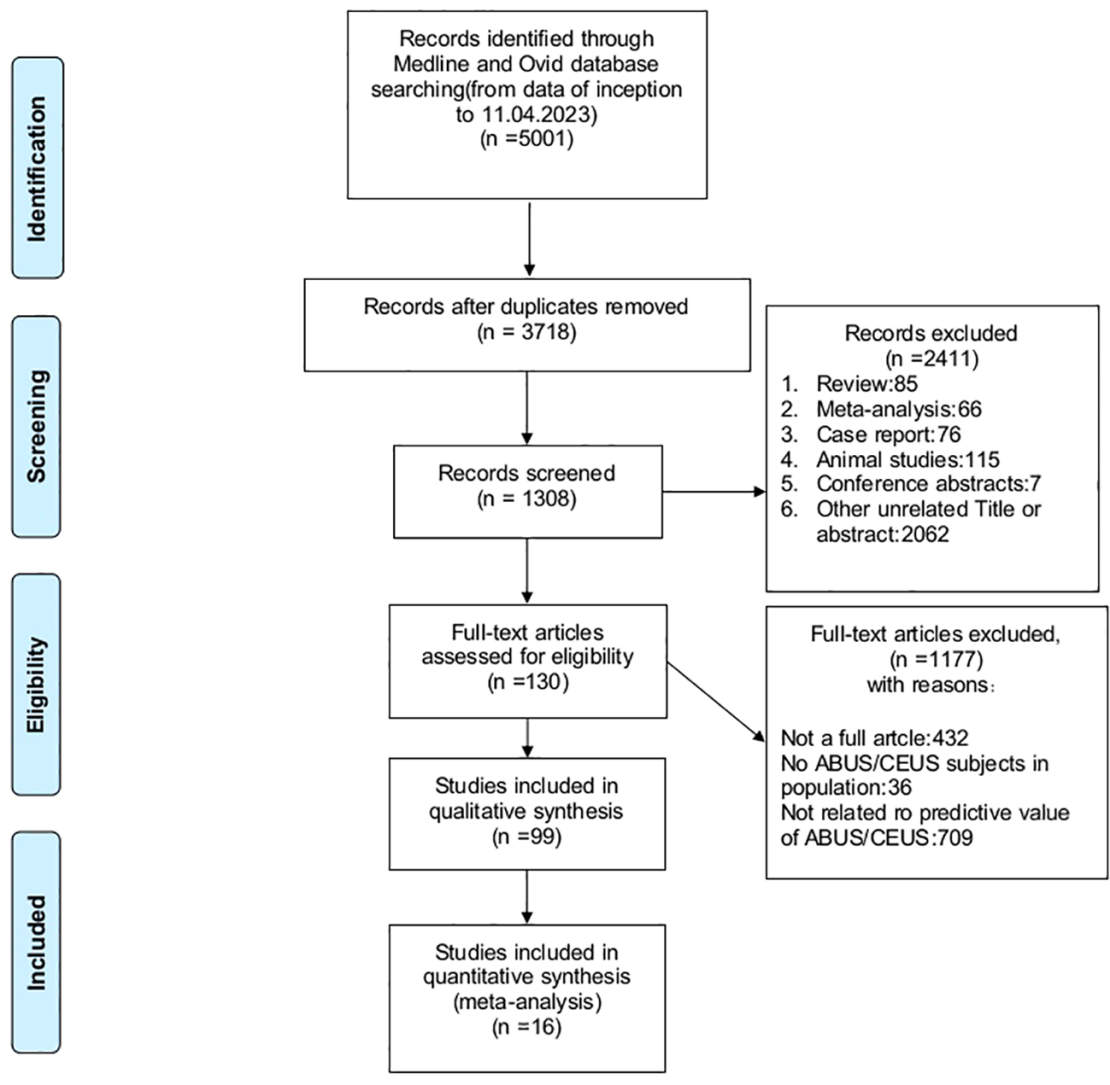
Literature screening process of the meta-analysis.

A total of 5001 studies were searched from four databases (PubMed, Embase, Cochrane Library and Web of Science). After elimination of 1283 duplicate records, 3718 related studies were included. Among these studies, 349 were omitted for being reviews, conference abstracts, meta-analyses, animal studies or case reports, whereas 2062 studies did not have relevant titles and abstracts. The full text of the remaining 130 studies were perused, and 1177 studies were excluded on account of imperfect data. The remaining 16 studies were extracted ultimately on the data extraction requirements. Eight studies used ABUS, and 8 used CEUS. The process of literature screening was performed in **Figure 1**. The basic characteristics of each study were plotted in **Table 1**.

**Table 1:** Basic characteristics of enrolled studies.

References	Year	Study	Region	Sample size	Age (years)	contrast agent		Diagnostic value		
						types	volume	sensitivity	specificity	AUC
Xi Lin (10)	2012	Prospective	China	95	16–78	_	_	100	95	_
Yuanming Xiao (11)	2015	Retrospective	China	273	18-72	_	_	28.95	100	0.7864
Hong-Yan Wang (12)	2012	Prospective	China	239	43.0 ± 12.5	_	_	95.3	80.5	0.948
LIN CHEN (13)	2013	Retrospective	China	219	16-71	_	_	92.5	86.2	_
Weixiang Liang (14)	2017	Retrospective	China	87	43.2 ± 14.5	_	_	81.8	85.2	0.879
Woo Jung Choi (15)	2014	Retrospective	Korea	1866	19-82	_	_	77.78	97.79	_
Jialin Liu (16)	2022	Prospective	China	431	16-82	_	_	92.16	87.05	0.901
Chaoli Xu (17)	2014	Retrospective	China	46	46 ± 1.6	_	_	100	77.8	_
Huiling He (18)	2023	Retrospective	China	26	23-76	SonoVue	4.8ml	85.71	68.42	0.74
Yingying Yuan (19)	2022	Prospective	China	108	53.37 ± 5.15	SonoVue	2.4ml	82.35	70	0.901
Zuopeng Ding (20)	2021	Retrospective	China	109	48.5 ± 10.4	SonoVue	4.8ml	88.46	74.19	0.9084
Natalia Caproni (21)	2010	Retrospective	Italy	43	28-85	SonoVue	5ml	91	72.73	_
Jing Du (22)	2008	Prospective	China	61	23-72	SonoVue	CnTI:3.6ml MFI:1.2ml	93.8	86.2	0.94
Yukio Miyamoto (23)	2014	Prospective	Japan	351	48.5 ± 12.3	Sonazoid	0.015 mL/kg	91.4	85.4	0.886
Daniela Stanzani (24)	2014	Prospective	São Paulo	70	18-78	Definity PESDA	Definity:0.01 mL/kg PESDA: 3ml	92	46.6	_
Caifeng Wan (25)	2012	Prospective	China	91	_	SonoVue	CnTI:3.6ml MFI:1.2ml	82.98	88.64	0.92

CnTI, contrast-tuned imaging; MFI, Microflow imaging; PESDA, perfluorocarbonexposed sonicated albumin.

### ABUS against breast cancer

**Figure 2:**
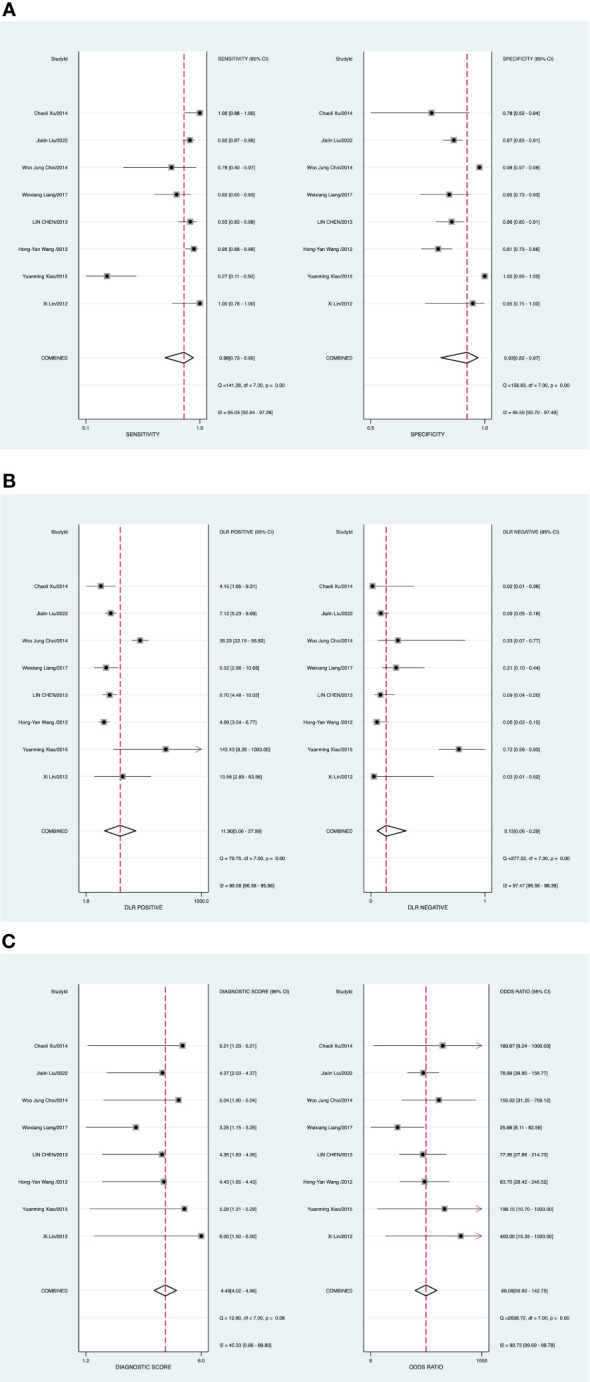
**(A)** Forest plot of sensitivity and specificity of automated breast ultrasound (ABUS) in the diagnosis of breast cancer. **(B)** Forest plot of the diagnosis likelihood ratio (DLR). **(C)** Forest plot of the diagnostic odds ratio (DOR).

The random-effects model was applied when the heterogeneity was greater than 50%. The combined sensitivity of ABUS against breast cancer was 0.88 [95% CI (0.73–0.95)], specificity was 0.93 [95% CI (0.82–0.97)], PLR was 11.9 [95% CI (5.1–28.0)], NLR was 0.13 [95% CI (0.06–0.29)], and DOR was 89.09 [95% CI (55.60–142.75)], indicating that ABUS had a high value in the screening of BC (**Figures 2A–C**).

### Publication bias and heterogeneity

Potential publication bias was assessed by the Deeks’ funnel plots in the process of detecting BC with ABUS. *P* value of 0.24 (**Supplementary Figure 1**) indicated no existing publication bias. There was one study out of the border, representing heterogeneity among included studies, as plotted in **Supplementary Figure 2**.

### Threshold effect

**Figure 3:**
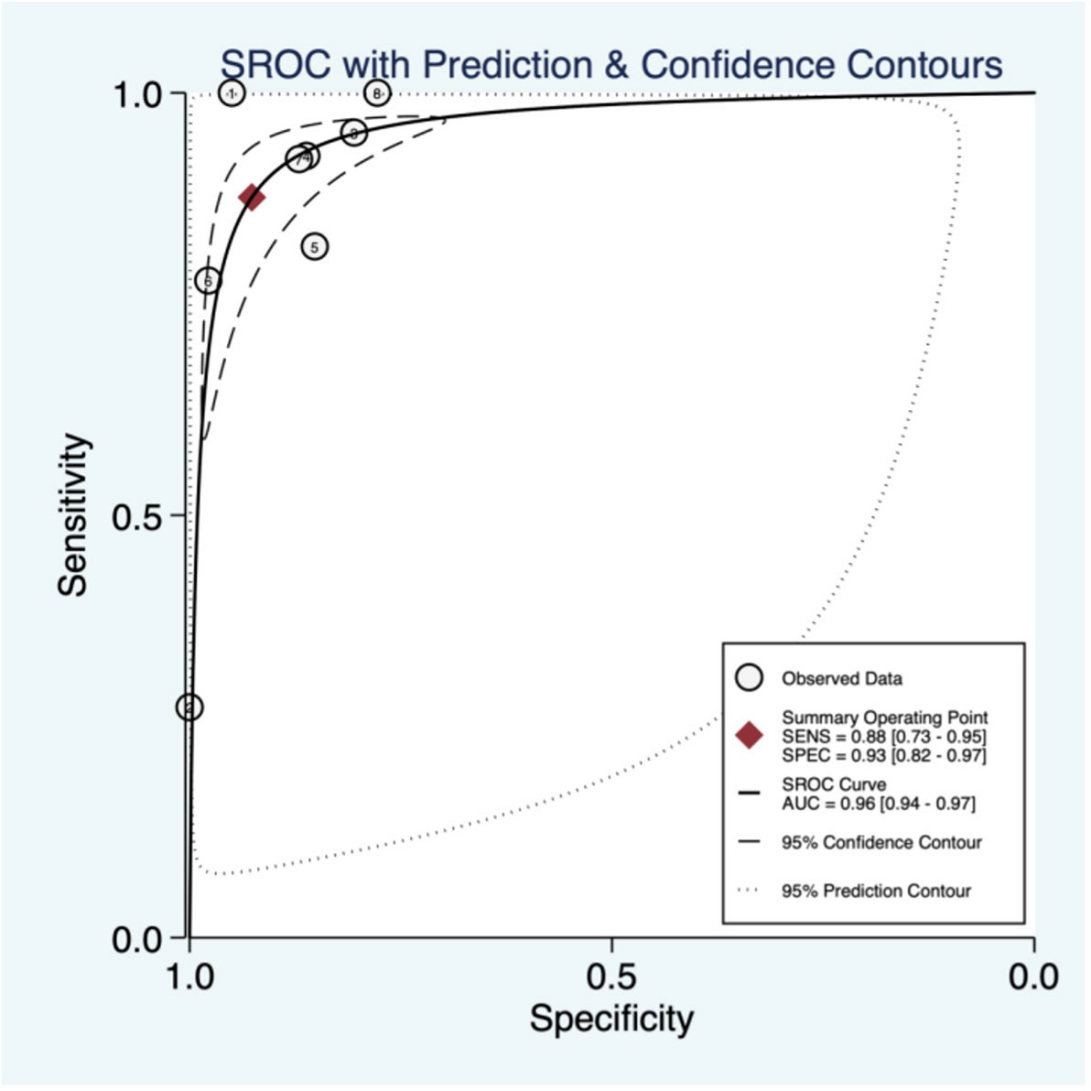
Summary of receiver operating characteristics of automated breast ultrasound (ABUS).

The threshold effect was assessed by the SROC curve plane test. The typical “shoulder arm” was absent, indicating the inexistence of the threshold effect. The area under the SROC curve (AUC) was 0.96 [95% CI (0.94–0.96)], indicating a high diagnostic value of ABUS (**Figure 3**).

### Pre-test probability, LR and post-test probability

**Figure 4:**
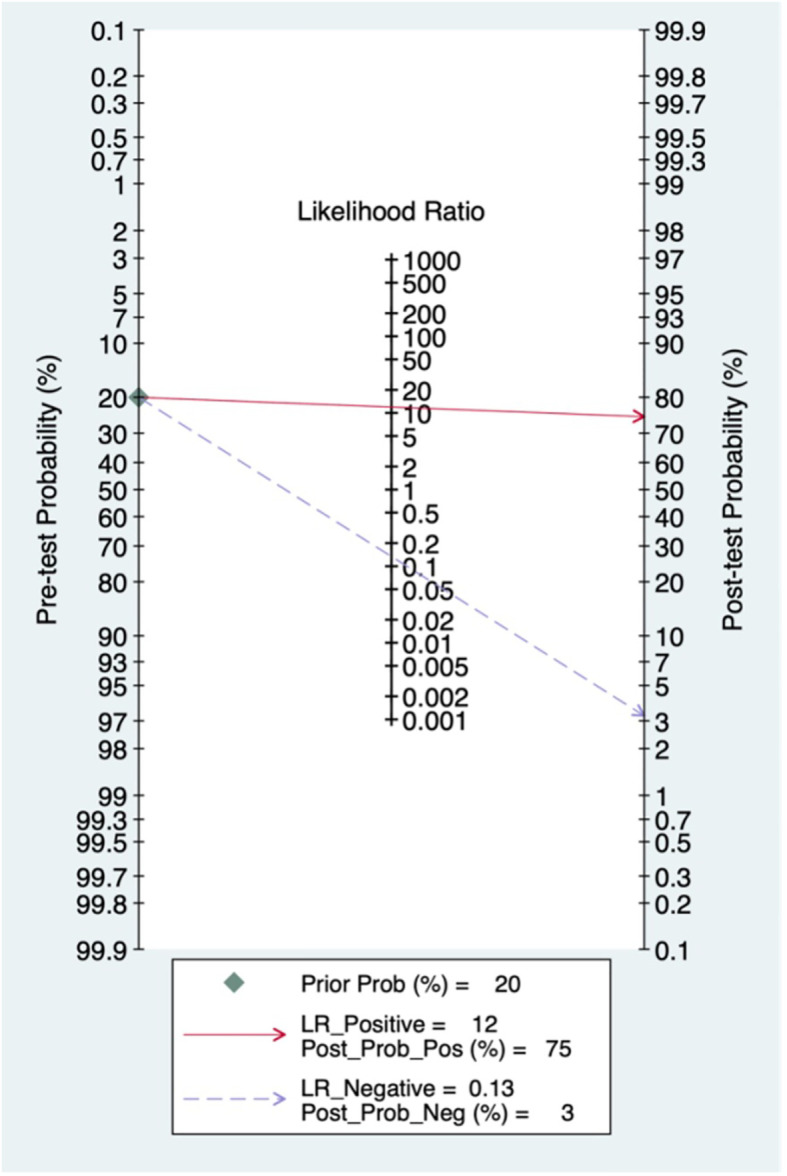
Fagan diagram of automated breast ultrasound (ABUS) in the diagnosis of breast cancer.

The relationships among the prior probability, the PLR, the NLR and the posterior probability were assessed via a Fagan graph. When the pre-test probability was set to 20%, the post-test probability of BC was 75%. Moreover, the positive likelihood ratio (PLR) was >10 (PLR = 12), and the negative likelihood ratio (NLR) was > 0.1 (NLR = 0.13), indicating that the ability to diagnose true positives was better (**Figure 4**).

### Meta-regression and subgroup analysis

Some factors, including a prospective design (prodesign), partial verification bias (fulverif), an adequate description of the study participants (subjdescr), report of method, a broad spectrum of diseases (brdspect), and whether the test results were assigned a value by a blind method, might be relevant to heterogeneity among these ABUS studies. The meta-regression analysis of the above-mentioned factors indicated that prodesign and blinding might be the source of heterogeneity of sensitivity (**Supplementary Figure 3**).

### CEUS against breast cancer

**Figure 5:**
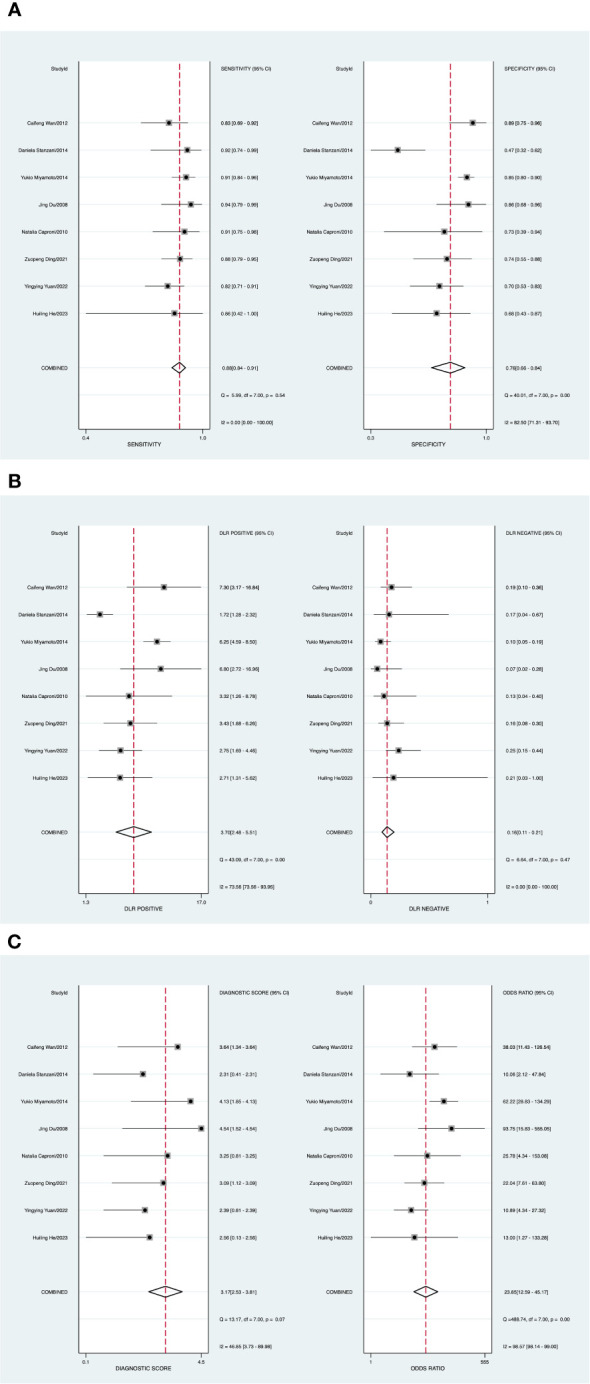
**(A)** Forest plot of sensitivity and specificity of contrast-enhanced ultrasound (CEUS) in the diagnosis of breast cancer. **(B)** Forest plot of diagnosis likelihood ratio (DLR). **(C)** Forest plot of the diagnostic odds ratio (DOR).

A random-effects model was applied when the heterogeneity was greater than 50%. The combined sensitivity of CEUS against breast cancer was 0.88 [95% CI (0.84–0.91)], specificity was 0.76 [95% CI (0.66–0.84)], PLR was 3.7 [95% CI (2.5–5.5)], NLR was 0.16 [95% CI (0.11–0.21)], and DOR was 23.85 [95% CI (12.59–45.17)], indicating that CEUS had a high value in the screening of BC (**Figures 5A–C**).

### Publication bias and heterogeneity

A *P* value of 0.20 (*P* > 0.05) (**Supplementary Figure 4**) indicated the absence of publication bias. There was one study outside of the border, representing heterogeneity among the included studies (**Supplementary Figure 5**).

### Threshold effect

**Figure 6:**
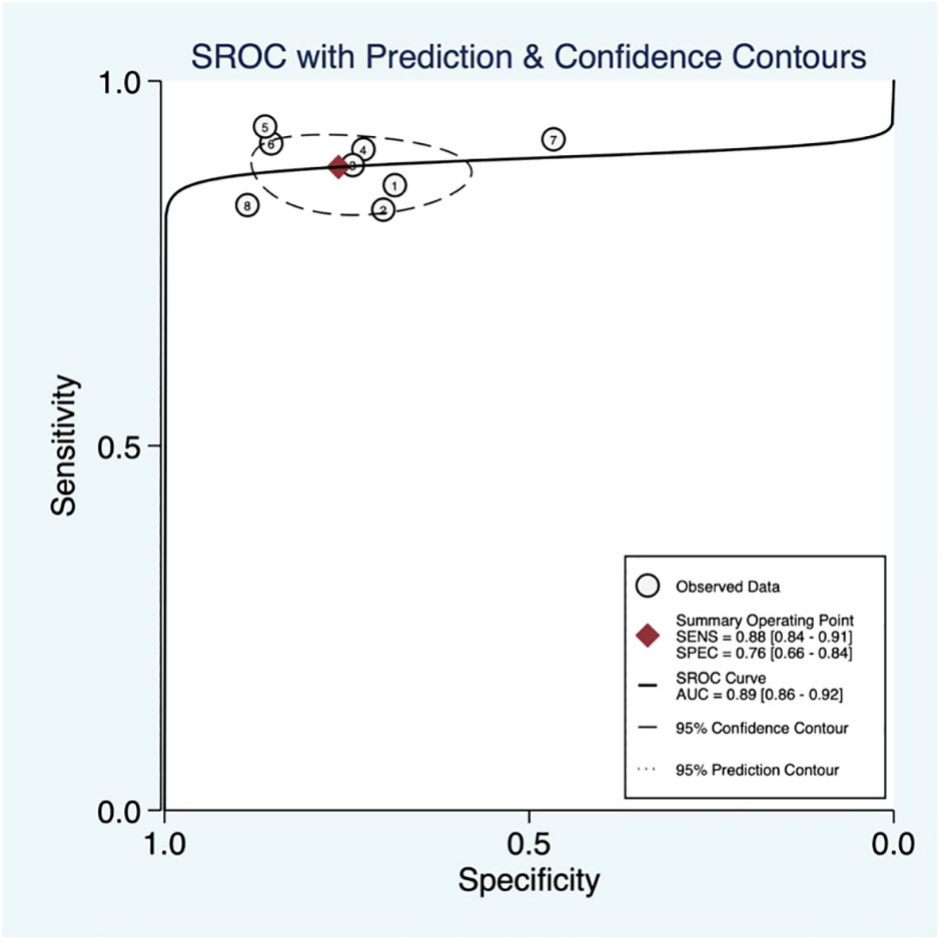
Summary of receiver operating characteristics of contrast-enhanced ultrasound (CEUS).

The threshold effect was assessed by the SROC curve plane test. The typical “shoulder arm” was absent, as revealed in **Figure 6**, indicating the inexistence of a threshold effect. The area under the SROC curve (AUC) was 0.89 [95% CI (0.86–0.92)], indicating a high diagnostic value of ABUS.

### Pre-test probability, LR and post-test probability

**Figure 7:**
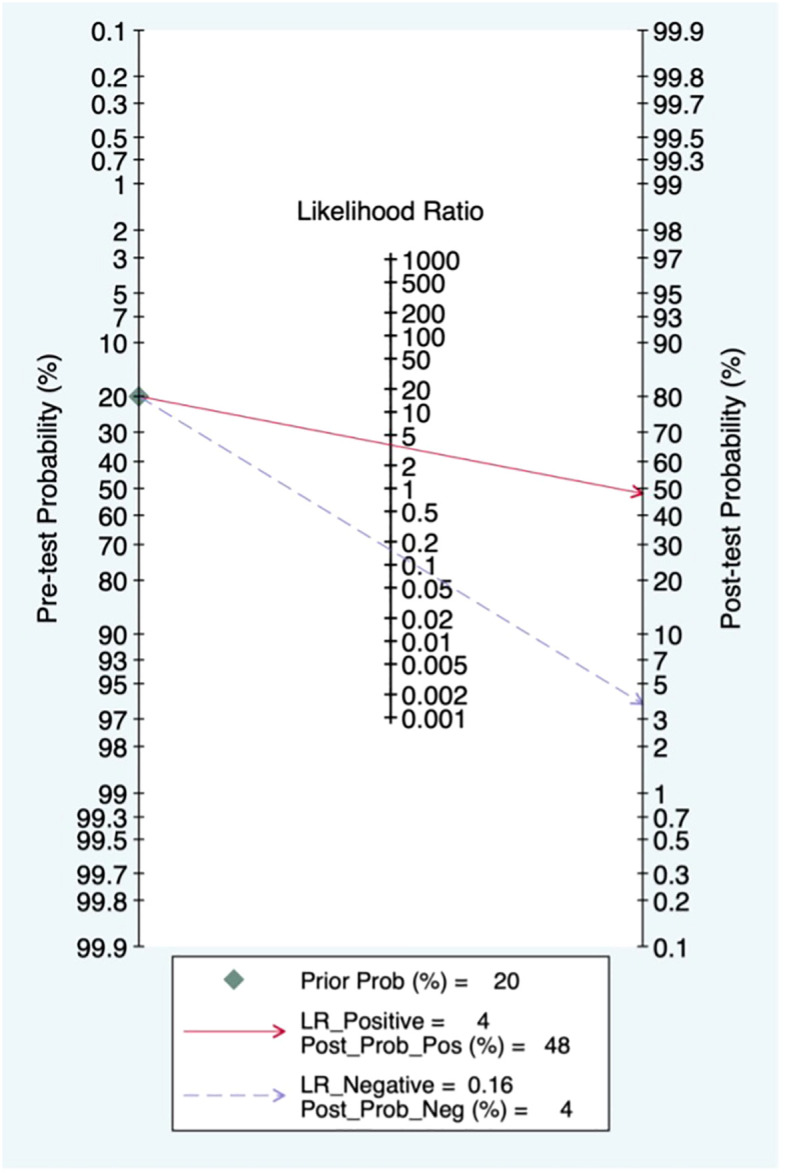
Fagan diagram of contrast-enhanced ultrasound (CEUS) in the diagnosis of heart sounds.

When the pre-test probability was set to 20%, the post-test probability of BC was 48%. Moreover, the positive likelihood ratio (PLR) was <10 (PLR = 4), and the negative likelihood ratio (NLR) was >0.1 (NLR = 0.16), indicating that the diagnosis could neither be confirmed nor excluded. The diagnostic value of CEUS in BC was limited, as shown in **Figure 7**.

### Meta-regression and subgroup analysis

The meta-regression analysis indicated that prodesign and blinding might be sources of heterogeneity of sensitivity, and no factors were related to sources of heterogeneity of specificity (**Supplementary Figure 6**).

### Comparison of ABUS and CEUS

A comparison of ABUS and CEUS was performed using ROC, sensitivity, and specificity analyses. Among them, ABUS had the best diagnostic value; details are shown in **Table 2**.

**Table 2:** Diagnostic performance of ABUS and CEUS.

Method	AUC	Sensitivity	Specificity	Prior *P*	PLR (%)	NLR(%)
ABUS	0.96	0.88	0.93	20	11.9	0.13
CEUS	0.89	0.88	0.76	20	3.7	0.16

ABUS, automated breast ultrasound; CEUS, contrast-enhanced ultrasound; PLR, the positive likelihood ratio; NLR, the negative likelihood ratio

## Discussion

The global incidence of BC is increasing each year (26). Early diagnosis can improve the prognosis significantly, especially when the lesions cannot be felt (27). Therefore, early diagnosis and symptomatic therapy in BC patients have weighty significance.

This systematic review and meta-analysis assessed the diagnostic efficiency of ABUS and CEUS in BC. A total of 16 studies, involving 4115 samples, were included in the analysis. Both ABUS and CEUS had a certain diagnostic value for breast cancer, as assessed by DOR. In addition, ABUS has a higher specificity and a larger AUC than CEUS. Moreover, ABUS improved the post-test probability to a greater extent than CEUS. The results showed that the diagnostic performance of ABUS was higher than that of CEUS. It should be noted that according to the search strategy, after screening by the inclusion and exclusion criteria, the final included literatures were mainly from Asian countries. Several studies has reported that for breast cancer dense breast as a risk varying from the lowest to highest sort of density by 4-6 folds, severally (28, 29). The breast density of women arguably in western countries are much lower than in Asian countries (30–33). This may be one reason that the final included literatures mainly focused on Asian countries.

ABUS is a time-saving method and a money-saving method. For breast cancers, the primary screening method is mammography. But its sensitivity is lower for dense breasts. Kim (34) found that mammography had a lower sensitivity in screening lesions of dense breasts as an independent risk factor for breast cancers. ABUS could become a supplementary diagnostic method to mammography when detecting masses in women with dense breasts (34). There are more and more studies for ABUS.

Vourtsis (35) claimed that a three-dimensional automated breast ultrasound system (3D ABUS) used high-frequency ultrasonic transducers and scanned most of the breast at once, which largely addressed the limitations of HHUS.

CEUS is a convenient imaging technique that allows patients to take a more appropriate position and shorter examination time than MRI, and CEUS can also be used in patients with MRI contraindications such as ferromagnetic metal implants. CEUS which is a high-performance, feasible, easy-to-implement, non-irradiating, accessible imaging method has proven to be a valuable complement to breast ultrasound (36). Hu (37) claimed that CEUS could display the features of breast lesions accurately and be helpful for selecting suspected malignant masses for surgery.

ABUS and CEUS also have their own limitations in the application process. ABUS has no ability to evaluate the condition of axillary nodes. Moreover, it still cannot guide the puncture biopsy. ABUS may miss lesions if there is a mass at the outer position of the breast. If the lotion in the ultrasound gel is not distributed homogeneously or even missing an area, air will enter the interspace between the transducer and the skin, inducing the inability to visualize the tissue beneath (38). In addition, the accuracy of CEUS used for detecting ductal carcinoma *in situ* (DCIS) and some rare types of BC is low (37).

The combination of the two may help to improve the ability to diagnose breast cancer. Quan et al. (39) indicated in the recently published literature that ABUS pooled with CEUS had higher precision in the analysis of BC and showed great application value in the judgment of breast cancer. In addition, Yongwei et al. (40) aimed to evaluate the role of ABUS and CEUS in the early prediction of the treatment response to neoadjuvant chemotherapy (NAC) in patients with BC and found that the CEUS-ABUS model could be used clinically to optimize the treatment of patients with breast cancer. However, the current number of studies on this topic is insufficient for a systematic review, which provides direction for our future research.

### Limitations

This study has several limitations. Firstly, because of the retrospective studies in this meta-analysis, there was likely to be subject selection bias. For example, most of the studies included were from Asia, especially China, which may cause bias of this research. Secondly, the relatively small sample sizes of the included studies may lead to overestimation of the diagnostic capacity. Thirdly, significant heterogeneity existing among the included reports could reduce the statistical efficiency. It is worth looking into further assessing the diagnostic power of CEUS and ABUS in a large-scale and prospective diagnostic study.

## Conclusions

The use of ABUS showed higher specificity and DOR for detecting BC compared with CEUS. ABUS is expected to further improve the diagnostic accuracy of breast cancer.

## Data availability statement

The original contributions presented in the study are included in the article/**Supplementary Material**. Further inquiries can be directed to the corresponding authors.

## Author contributions

HZ: Writing – original draft. JH: Writing – original draft. RM: Writing – original draft. FL: Writing – original draft. FX: Writing – review & editing. MH: Writing – review & editing.

## Supplementary material

Supplementary Figure 1 Deeks’ funnel plot.

Supplementary Figure 2 Bivariate boxplot.

Supplementary Figure 3 Multiple univariate meta-regression and subgroup analysis. Prospective design: prodesign; fulverif: partial verification bias; subjdescr: adequate description of study participants; brdspect: broad spectrum of disease.

Supplementary Figure 4 Deeks’ funnel plot.

Supplementary Figure 5 Bivariate boxplot.

Supplementary Figure 6 Multiple univariate meta-regression and subgroup analysis. Prospective design: prodesign; fulverif: partial verification bias; subjdescr: adequate description of study participants; brdspect: broad spectrum of disease.

## References

[1] Sung H Ferlay J Siegel RL Laversanne M Soerjomataram I Jemal A . Global Cancer Statistics 2020: GLOBOCAN estimates of incidence and mortality worldwide for 36 cancers in 185 countries. CA Cancer J Clin (2021) 71(3):209–49. doi: 10.3322/caac.21660 33538338

[2] Yi JC Syrjala KL . Anxiety and depression in cancer survivors. Med Clinics North America (2017) 101(6):1099–+. doi: 10.1016/j.mcna.2017.06.005 PMC5915316 28992857

[3] Altice CK Banegas MP Tucker-Seeley RD Yabroff KR . Financial hardships experienced by cancer survivors: A systematic review. J Natl Cancer Inst (2017) 109(2). doi: 10.1093/jnci/djw205 PMC6075571 27754926

[4] Giaquinto AN Sung H Miller KD Kramer JL Newman LA Minihan A . Breast cancer statistics, 2022. CA Cancer J Clin (2022) 72(6):524–41. doi: 10.3322/caac.21754 36190501

[5] Aminawung JA Hoag JR Kyanko KA Xu X Richman IB Busch SH . Breast cancer supplemental screening: Women’s knowledge and utilization in the era of dense breast legislation. Cancer Med (2020) 9(15):5662–71. doi: 10.1002/cam4.3218 PMC7402830 32537899

[6] Ohnuki K Tohno E Tsunoda H Uematsu T Nakajima Y . Overall assessment system of combined mammography and ultrasound for breast cancer screening in Japan. Breast Cancer (2021) 28(2):254–62. doi: 10.1007/s12282-020-01203-y PMC7925504 33389614

[7] Chan SW Cheung PS Chan S Lau SS Wong TT Ma M . Benefit of ultrasonography in the detection of clinically and mammographically occult breast cancer. World J Surg (2008) 32(12):2593–8. doi: 10.1007/s00268-007-9273-2 17960454

[8] Guo R Lu G Qin B Fei B . Ultrasound imaging technologies for breast cancer detection and management: A review. Ultrasound Med Biol (2018) 44(1):37–70. doi: 10.1016/j.ultrasmedbio.2017.09.012 29107353 PMC6169997

[9] Xu H Xu GL Li XD Su QH Dong CZ . Correlation between the contrast-enhanced ultrasound image features and axillary lymph node metastasis of primary breast cancer and its diagnostic value. Clin Trans Oncol (2021) 23(1):155–63. doi: 10.1007/s12094-020-02407-6 32488804

[10] Lin X Wang J Han F Fu J Li A . Analysis of eighty-one cases with breast lesions using automated breast volume scanner and comparison with handheld ultrasound. Eur J Radiol (2012) 81(5):873–8. doi: 10.1016/j.ejrad.2011.02.038 21420814

[11] Xiao Y Zhou Q Chen Z . Automated breast volume scanning versus conventional ultrasound in breast cancer screening. Acad Radiol (2015) 22(3):387–99. doi: 10.1016/j.acra.2014.08.013 25620036

[12] Wang H-Y Jiang Y-X Zhu Q-L Zhang J Dai Q Liu H . Differentiation of benign and Malignant breast lesions: A comparison between automatically generated breast volume scans and handheld ultrasound examinations. Eur J Radiol (2012) 81(11):3190–200. doi: 10.1016/j.ejrad.2012.01.034 22386134

[13] Chen L Chen Y Diao XH Fang L Pang Y Cheng AQ . Comparative study of automated breast 3-D ultrasound and handheld B-mode ultrasound for differentiation of benign and Malignant breast masses. Ultrasound Med Biol (2013) 39(10):1735–42. doi: 10.1016/j.ultrasmedbio.2013.04.003 23849390

[14] Liang W Yu J Xie Y Jiang L Zhou X Feng S . The differential diagnosis of ultrasonic imaging by automated breast volume scanning in breast cancer. Eur J Gynaecological Oncol (2018) 39(4):548–53. doi: 10.12892/ejgo4131.2018

[15] Choi WJ Cha JH Kim HH Shin HJ Kim H Chae EY . Comparison of automated breast volume scanning and hand- held ultrasound in the detection of breast cancer: an analysis of 5,566 patient evaluations. Asian Pac J Cancer Prev (2014) 15(21):9101–5. doi: 10.7314/APJCP.2014.15.21.9101 25422185

[16] Liu J Zhou Y Wu J Li P Liang X Duan H . Diagnostic performance of combined use of automated breast volume scanning & hand-held ultrasound for breast lesions. Indian J Med Res (2021) 154(2):347–54. doi: 10.4103/ijmr.IJMR_836_19 PMC9131766 35295015

[17] Xu C Wei S Xie Y Guan X Fu N Huang P . Combined use of the automated breast volume scanner and the US elastography for the differentiation of benign from Malignant lesions of the breast. BMC Cancer (2014) 14:798. doi: 10.1186/1471-2407-14-798 25366878 PMC4228072

[18] He H Wu X Jiang M Xu Z Zhang X Pan J . Diagnostic accuracy of contrast-enhanced ultrasound synchronized with shear wave elastography in the differential diagnosis of benign and Malignant breast lesions: a diagnostic test. Gland Surg (2023) 12(1):54–66. doi: 10.21037/gs-22-684 36761482 PMC9906099

[19] Yuan Y Xu M Ren Y He L Chen J Sun L . Clinical value of contrast-enhanced ultrasound in breast cancer diagnosis. Comput Math Methods Med (2022) 2022:2017026. doi: 10.1155/2022/2017026 36105240 PMC9467778

[20] Ding Z Liu W He N Ma X Fu L Ye L . Value of ultrasound elastography combined with contrast-enhanced ultrasound and micro-flow imaging in differential diagnosis of benign and Malignant breast lesions. Am J Transl Res (2021) 13(12):13941–9. PMC8748137 35035735

[21] Caproni N Marchisio F Pecchi A Canossi B Battista R D’Alimonte P . Contrast-enhanced ultrasound in the characterisation of breast masses: utility of quantitative analysis in comparison with MRI. Eur Radiol (2010) 20(6):1384–95. doi: 10.1007/s00330-009-1690-1 20033178

[22] Du J Li FH Fang H Xia JG Zhu CX . Microvascular architecture of breast lesions: evaluation with contrast-enhanced ultrasonographic micro flow imaging. J Ultrasound Med (2008) 27(6):833–42; quiz 44. doi: 10.7863/jum.2008.27.6.833 18499843

[23] Miyamoto Y Ito T Takada E Omoto K Hirai T Moriyasu F . Efficacy of sonazoid (perflubutane) for contrast-enhanced ultrasound in the differentiation of focal breast lesions: phase 3 multicenter clinical trial. AJR Am J Roentgenol (2014) 202(4):W400–7. doi: 10.2214/AJR.12.10518 24660739

[24] Stanzani D Chala LF de Barros N Cerri GG Chammas MC . Can Doppler or contrast-enhanced ultrasound analysis add diagnostically important information about the nature of breast lesions? Clinics (2014) 69(2):87–92. doi: 10.6061/clinics/2014(02)03 24519198 PMC3912319

[25] Wan C Du J Fang H Li F Wang L . Evaluation of breast lesions by contrast enhanced ultrasound: qualitative and quantitative analysis. Eur J Radiol (2012) 81(4):e444–50. doi: 10.1016/j.ejrad.2011.03.094 21612882

[26] Tao Z Shi A Lu C Song T Zhang Z Zhao J . Breast cancer: epidemiology and etiology. Cell Biochem Biophys (2015) 72(2):333–8. doi: 10.1007/s12013-014-0459-6 25543329

[27] Alkabban FM Ferguson T . Breast Cancer. Treasure Island (FL: StatPearls (2023).

[28] McCormack VA Silva I . Breast density and parenchymal patterns as markers of breast cancer risk: A meta-analysis. Cancer Epidemiol Biomarkers Prev (2006) 15(6):1159–69. doi: 10.1158/1055-9965.EPI-06-0034 16775176

[29] McLean KE Stone J . Role of breast density measurement in screening for breast cancer. Climacteric (2018) 21(3):214–20. doi: 10.1080/13697137.2018.1424816 29447010

[30] Li J Zhang B-N Fan J-H Pang Y Zhang P Wang S-L . A Nation-Wide multicenter 10-year (1999-2008) retrospective clinical epidemiological study of female breast cancer in China. BMC Cancer (2011) 11. doi: 10.1186/1471-2407-11-364 PMC3178543 21859480

[31] Park B Cho HM Lee EH Song S Suh M Choi KS . Does breast density measured through population-based screening independently increase breast cancer risk in Asian females? Clin Epidemiol (2018) 10:61–70. doi: 10.2147/CLEP.S144918 29343988 PMC5749627

[32] Shen S Zhou Y Xu Y Zhang B Duan X Huang R . A multi-centre randomised trial comparing ultrasound vs mammography for screening breast cancer in high-risk Chinese women. Br J Cancer (2015) 112(6):998–1004. doi: 10.1038/bjc.2015.33 25668012 PMC4366890

[33] Stomper PC Dsouza DJ DiNitto PA Arredondo MA . Analysis of parenchymal density on mammograms in 1353 women 25-79 years old. Am J Roentgenol (1996) 167(5):1261–5. doi: 10.2214/ajr.167.5.8911192 8911192

[34] Kim SH Kim HH Moon WK . Automated breast ultrasound screening for dense breasts. Korean J Radiol (2020) 21(1):15–24. doi: 10.3348/kjr.2019.0176 31920025 PMC6960307

[35] Vourtsis A . Three-dimensional automated breast ultrasound: Technical aspects and first results. Diagn Interventional Imaging (2019) 100(10):579–92. doi: 10.1016/j.diii.2019.03.012 30962169

[36] Boca I Dudea SM Ciurea AI . Contrast-enhanced ultrasonography in the diagnosis and treatment modulation of breast cancer. J Personalized Med (2021) 11(2). doi: 10.3390/jpm11020081 PMC7912589 33573122

[37] Hu Q Wang XY Zhu SY Kang LK Xiao YJ Zheng HY . Meta-analysis of contrast-enhanced ultrasound for the differentiation of benign and Malignant breast lesions. Acta Radiologica (2015) 56(1):25–33. doi: 10.1177/0284185113517115 24436445

[38] Luczynska E Pawlak M Popiela T Rudnicki W . The role of ABUS in the diagnosis of breast cancer. J Ultrasonogr (2022) 22(89):76–85. doi: 10.15557/JoU.2022.0014 PMC9231518 35811591

[39] Yuan Q Song C Tian Y Chen N He X Wang Y . Diagnostic significance of 3D automated breast volume scanner in a combination with contrast-enhanced ultrasound for breast cancer. BioMed Res Int (2022) 2022. doi: 10.1155/2022/3199884 PMC9365610 35968241

[40] Xie Y Chen Y Wang Q Li B Shang H Jing H . Early prediction of response to neoadjuvant chemotherapy using quantitative parameters on automated breast ultrasound combined with contrast-enhanced ultrasound in breast cancer. Ultrasound Med Biol (2023) 49(7):1638–46. doi: 10.1016/j.ultrasmedbio.2023.03.017 37100671

