# Supplementary figures and images for: A systematic review and meta-analysis comparing the diagnostic capability of automated breast ultrasound and contrast-enhanced ultrasound in breast cancer
Source: Front Oncol. 2024 Jan 9;13:1305545. doi: 10.3389/fonc.2023.1305545 (PMC10803446; doi:10.3389/fonc.2023.1305545)

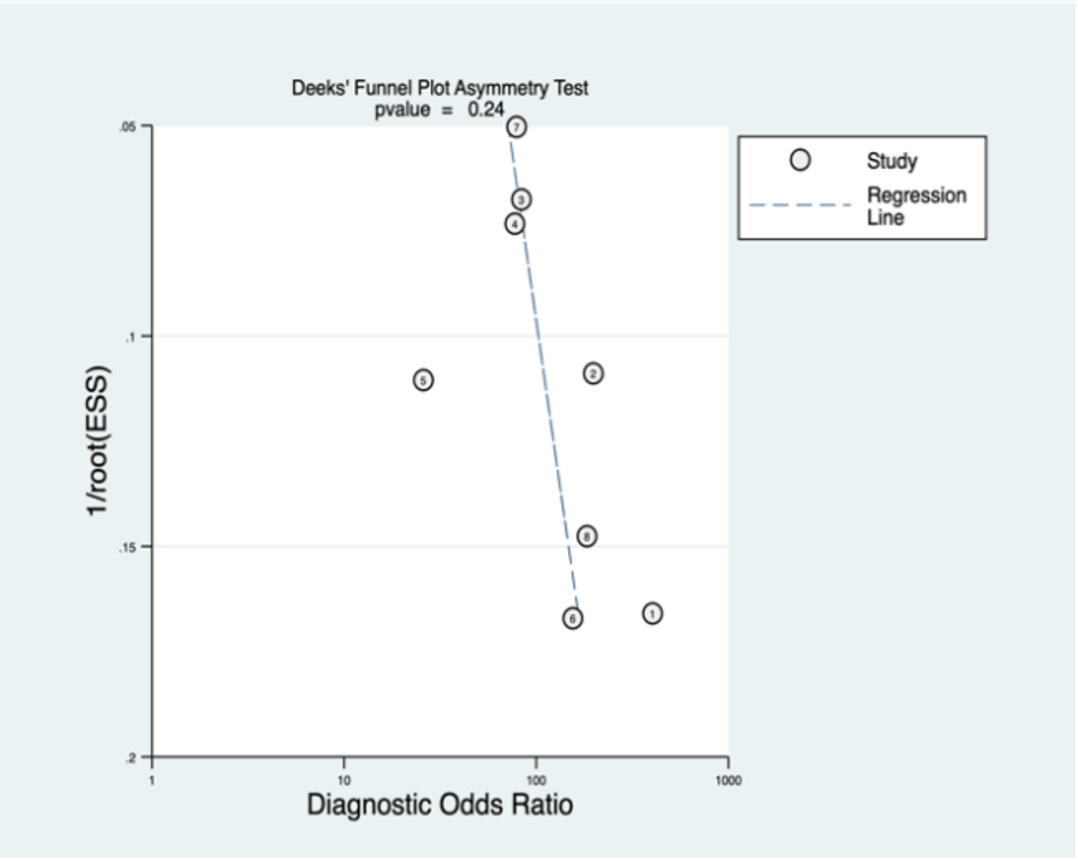

Supplement: Supplementary Figure 1 — Deeks’ funnel plot. [file Image_1.png]

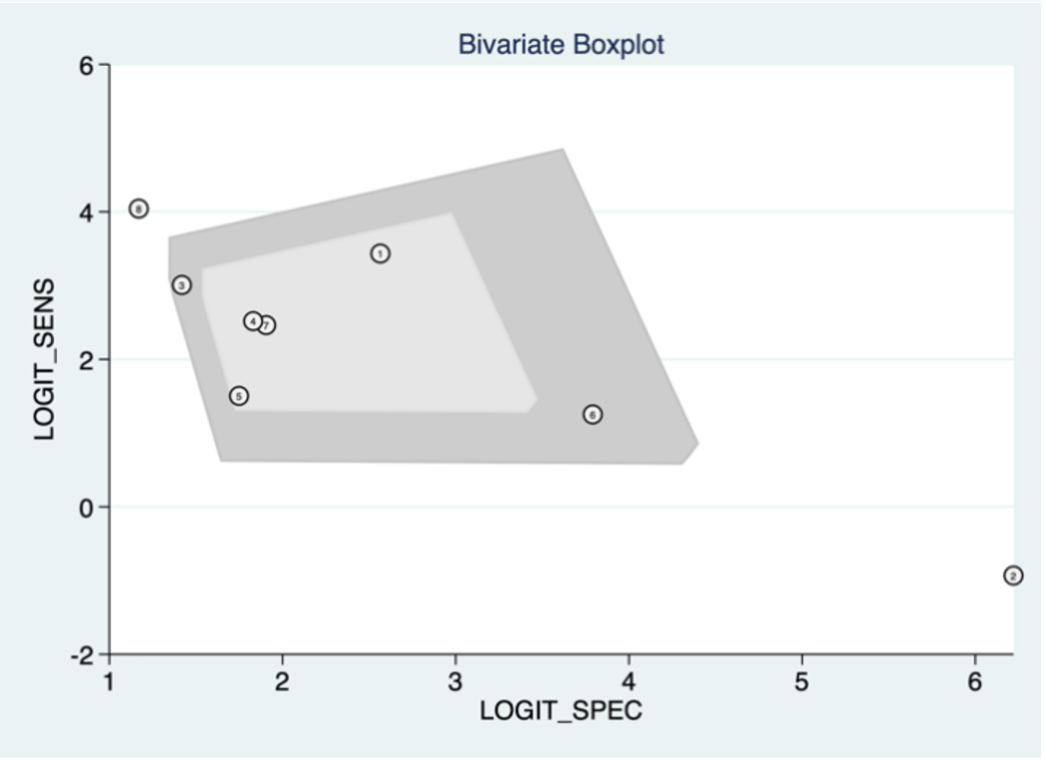

Supplement: Supplementary Figure 2 — Bivariate boxplot. [file Image_2.png]

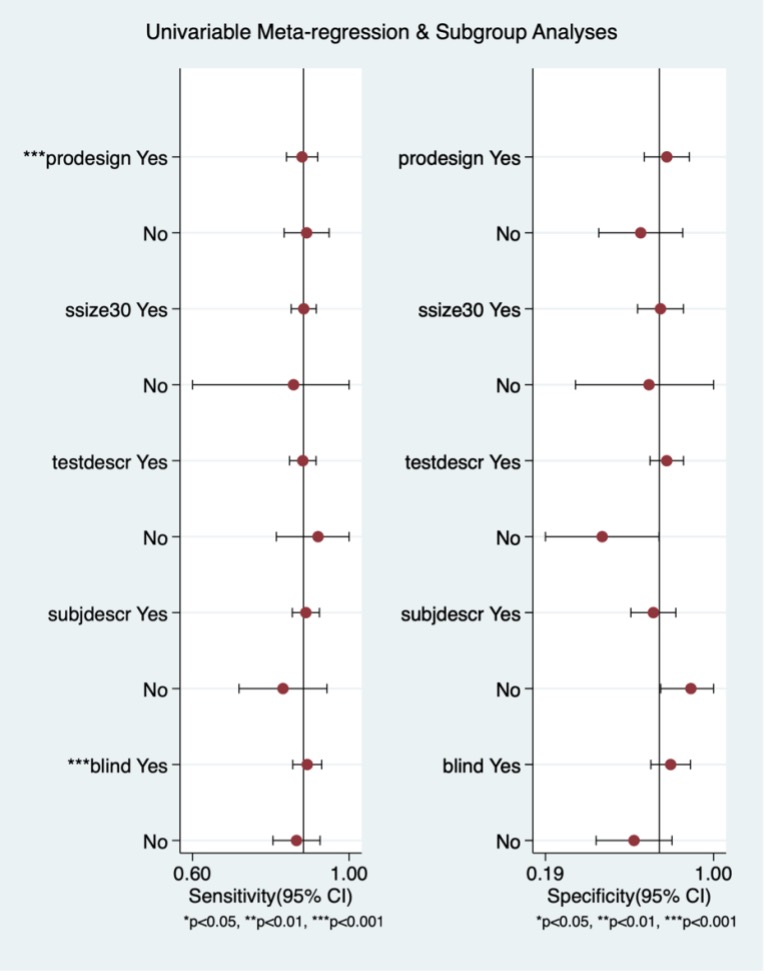

Supplement: Supplementary Figure 3 — Multiple univariate meta-regression and subgroup analysis. Prospective design: prodesign; fulverif: partial verification bias; subjdescr: adequate description of study participants; brdspect: broad spectrum of disease. [file Image_3.jpeg]

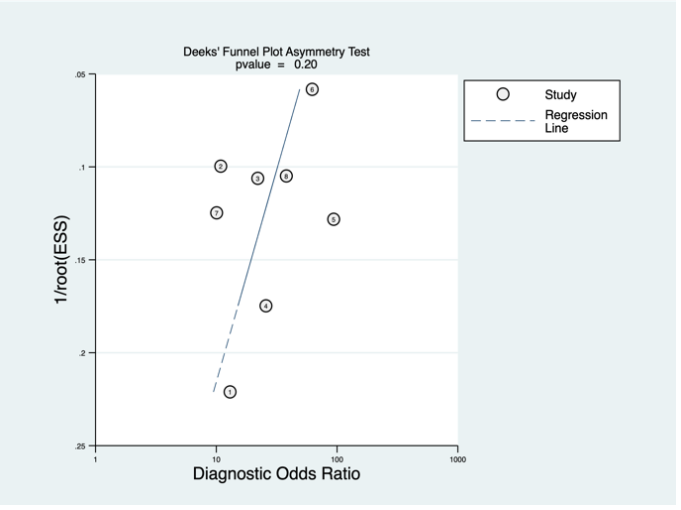

Supplement: Supplementary Figure 4 — Deeks’ funnel plot. [file Image_4.png]

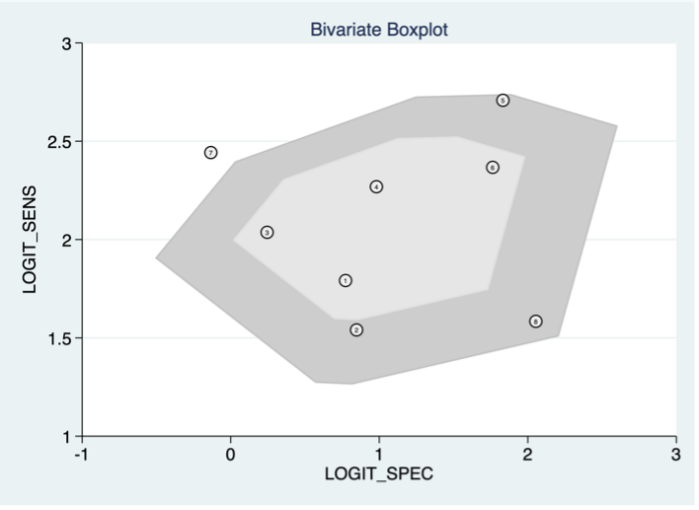

Supplement: Supplementary Figure 5 — Bivariate boxplot. [file Image_5.png]

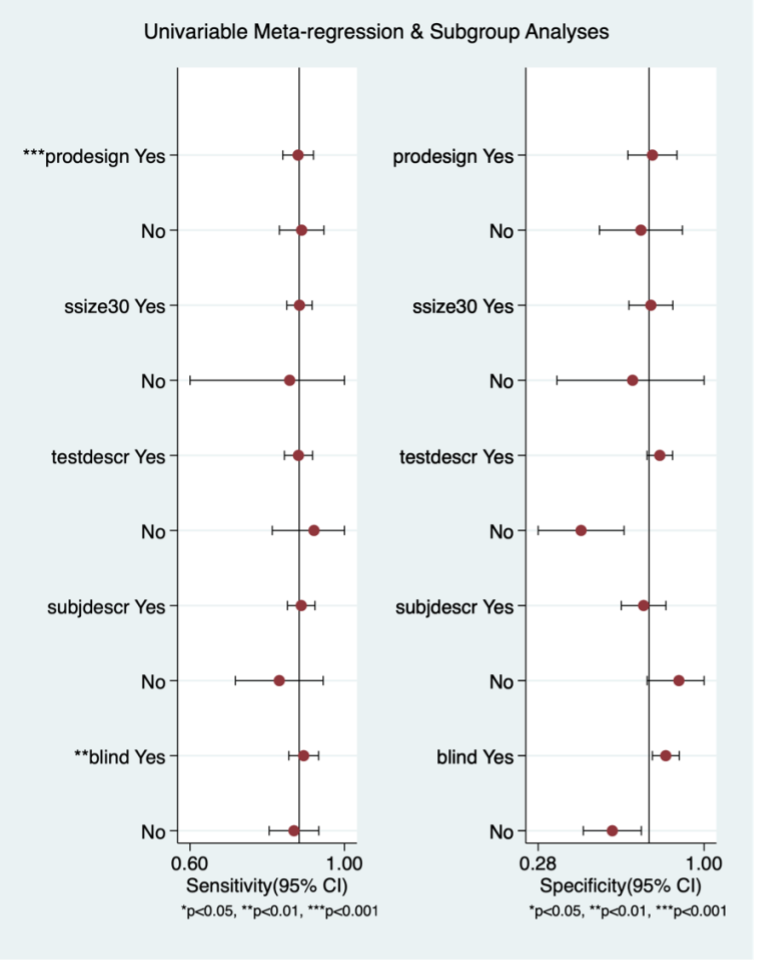

Supplement: Supplementary Figure 6 — Multiple univariate meta-regression and subgroup analysis. Prospective design: prodesign; fulverif: partial verification bias; subjdescr: adequate description of study participants; brdspect: broad spectrum of disease. [file Image_6.png]
